# Active and passive organic carbon fluxes during a bloom in the Southern Ocean (South Georgia)

**DOI:** 10.1038/s41597-024-04151-w

**Published:** 2024-12-18

**Authors:** William Major, Sarah L. C. Giering, Joanna Ainsworth, Anna Belcher, Sabena Blackbird, Martin Bridger, Nathan Briggs, Filipa Carvalho, Louis Clément, Kathryn Cook, Cynthia Dumousseaud, Benoit Espinola, Claire Evans, Sophie Fielding, Manuela Hartmann, Stephanie Henson, Morten Iversen, Konstadinos Kiriakoulakis, Richard Lampitt, Elisa Lovecchio, Adrian Martin, Dan Mayor, Mark Moore, Katsiaryna Pabortsava, Corinne Pebody, Kate Peel, Calum Preece, Alex Poulton, Rachel Rayne, Kevin Saw, Mark Stinchcombe, Gabriele Stowasser, Geraint A. Tarling, Sandy Thomalla, María Villa-Alfageme, George A. Wolff, Richard Sanders

**Affiliations:** 1 National Oceanography Centre, Southampton, UK; 2 University of Southampton, Southampton, UK; 3 British Antarctic Survey, Cambridge, UK; 4 University of Liverpool, Liverpool, UK; 5 University of Exeter, Exeter, UK; 6 Finnish Meteorological Institute, Helsinki, Finland; 7 University of Bremen, Bremen, Germany; 8 Liverpool John Moores University, Liverpool, UK; 9 Heriot-Watt University, Edinburgh, UK; 10 Council for Scientific and Industrial Research, Stellenbosch, South Africa; 11 University of Sevilla, Sevilla, Spain; 12 Norwegian Research Centre, Bergen, Norway; 13 Present Address: Centre for Ecology and Hydrology, Wallingford, UK

**Keywords:** Carbon cycle, Marine chemistry, Element cycles

## Abstract

The Controls Over Mesopelagic Interior Carbon Storage (COMICS) cruise DY086 took place aboard the RRS Discovery in the South Atlantic during November and December, 2017. Physical, chemical, biogeochemical and biological data were collected during three visits to ocean observatory station P3, off the coast of South Georgia, during an austral spring bloom. A diverse range of equipment including CTD-rosette, Acoustic Doppler Current Profiler (ADCP), net deployments, marine snow catchers (MSCs), Stand Alone Pump System (SAPS) and PELAGRA Sediment Traps were used to produce a comprehensive, high-quality dataset. The data can provide excellent insight into regional biological carbon pump (BCP) processes; it is recommended for use by observational scientists and modellers to enhance understanding of ecosystem interactions relating to mesopelagic carbon storage.

## Background & Summary

The ‘biological carbon pump’ (BCP) describes biogeochemical processes that contribute to organic carbon sequestration in the ocean. Organic matter originates from euphotic zone primary production and is transported to depth where it is remineralised. The BCP is a major control on Earth’s climate and models suggest it moderates atmospheric carbon dioxide levels by ~200 ppm^1^ relative to pre-industrial levels. Several processes that contribute to the vertical transfer of organic matter have been identified in the literature, including: the gravitational pump, the mesopelagic migrant pump, the seasonal lipid pump, the mixed-layer pump, the large-scale physical pump and the eddy-subduction pump^2^. Quantifying BCP processes simultaneously is difficult because a diverse range of scientific equipment is required and because of substantial temporal and spatial variability. However, synchronous measurements are essential if proportional contributions from individual BCP facets are to be accurately distinguished.

**Fig. 1:**
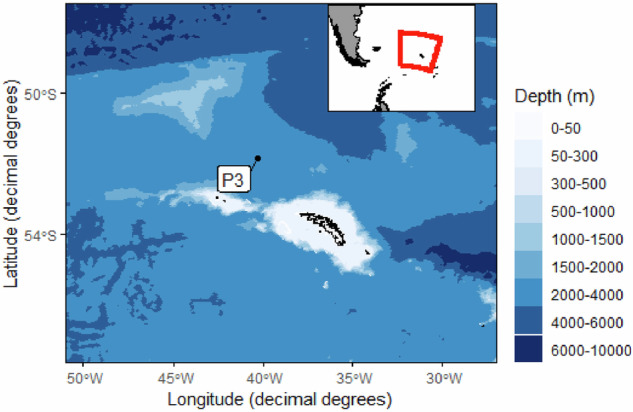
A map of the collection region: Long-term observation station ‘P3’ (52.4 °S, 40.1°W) in the vicinity of South Georgia.

The Controls Over Mesopelagic Interior Carbon Storage (COMICS) project aimed to gain a greater understanding of transfer efficiency of organic carbon through the mesopelagic ocean^3^. Data collection was planned for site P3 (52.40 °S, 40.06 °W) in the South Atlantic, Northwest of South Georgia (Fig. 1). P3 is a long-term study site operated by the British Antarctic Survey (BAS) since 2006^4^. P3 is situated in an area that experiences elevated primary production due to island-derived iron fertilisation. Gravitational carbon export and export efficiency are higher relative to another BAS study site (P2) situated 300 km to the south^4^. Further, low mesoscale variability means that the influence of the eddy-subduction pump is diminished; upwelling means that the large-scale physical pump is weak in the region^5^. Therefore, P3 permits a focus on the gravitational, mesopelagic migrant and mixed-layer pumps.

Few examples exist in the literature of simultaneous measurements of the gravitational, mesopelagic migrant and mixed-layer pumps. Datasets containing these parameters can be integrated by models and used to enhance our understanding of how biological interactions affect carbon storage. In particular, the ecosystem services provided by mesopelagic fishes are of great interest due to the growing commercial viability of their exploitation^6^. Observational and model studies suggest the impact of mesopelagic fishes on carbon storage is significant^7,8^. Therefore, it is vital that the contribution of mesopelagic fishes to carbon storage is elucidated before stocks are affected.

To address the lack of simultaneous vertical organic matter flux measurements, we present data collected from P3 during the COMICS cruise in November and December, 2017. Dissolved organic carbon (DOC) and particulate organic carbon (POC) concentration and vertical flux data accompanied by acoustic- and net-derived active flux measurements allow simultaneous quantification of the relevant pumps. Most data presented here are held by British Oceanographic Data Centre (BODC); they have not been curated and can be downloaded as individual parameters. Data essential to investigating the BCP that are described elsewhere in the literature are outlined in the Methods section; the majority remained unavailable prior to this work. The PANGAEA dataset brings all the data together in five files to provide an opportunity to investigate BCP processes and their related ecosystem functions. The data are available in one convenient location for users and follow the FAIR principles^9^.

## Methods

Data collection was carried out in November and December, 2017 as part of the COMICS project^3^. Site P3 was visited three times during the cruise with each visit approximately 7–8 days in duration: P3A (15–22nd November), P3B (29th November – 5th December), and P3C (9–15th December). Some data along with their collection and analysis methodologies have been described previously in the literature and are referenced herein. Details of sensors and equipment used for data collection are included with the data. All data were imported into R (version 4.3.1; see Code Availability for further information).

*‘Ship-based CTD profile data’* (Table 1) contains data from the two CTD-rosettes that were used during the campaign: one made of stainless steel and the other made of titanium suitable for trace metal sampling (CTD Events 4, 7, 15, 19, 24, 29). CTD Event numbers 1, 11, 12, 13, 14 and 25 were removed as these deployments were not made at site P3. Sensors attached to each of the CTD-rosettes included: Sea-Bird SBE sensors (two 3Plus temperature; 4 C conductivity; 43 dissolved oxygen); Paroscientific Digiquartz with TC Depth sensor; WETLabs ECO-BB OBS Scattering Meter; Biospherical LICOR Photosynthetically Active Radiation (PAR) sensor; WET Labs C-Star Transmissometer; Chelsea Aquatracka MKIII fluorometer. Temperature, salinity, dissolved oxygen sensor and chlorophyll-a data were calibrated against in situ bottle measurements. Measurements from bottle samples also include nitrate (n = 224), phosphate (n = 223) and silicate (n = 224) which were determined by colorimetric analysis^10^; the method for POC bottle samples (n = 77) has been previously described in the literature along with other discrete POC samples (presented in ‘*Discrete POC concentration and flux data’;* Table 3)^11^. Other parameters in ‘*Ship-based CTD profile data’* that have previously been described in the literature include: net primary productivity (NPP)^12^; turbulence, dissolved organic carbon (DOC, n = 5) and DOC flux (n = 1)^13^; ambient leucine assimilation (n = 31) and bacterial cell count^14^ (n = 34); chlorophyll-a^10^. ‘*Ship-based meteorological data’* (Table 2) contains the ship’s weather presented in every minute for each P3 visit; anemometer data was not included because of inconsistencies identified by BODC.

**Table 1:** Parameters included in ship-based CTD profile data file.

Parameter	Column header	Unit	No. profiles
Event	Event	N/A	—
DateTime	Date/Time	YYYY-MM-DD HH:mm:ss	—
Latitude	Latitude	degrees North	—
Longitude	Longitude	degrees East	—
Site	Site	N/A	—
Depth	Depth water [m] (Barometer, Paroscientific, Di…)	metres	—
Temperature	Temp [°C] (Temperature sensor, SEA-BIRD…)	degrees C	27
Salinity	Sal (PSU, Conductivity sensor, SEA…)	PSU	27
SigmaTheta	Sigma-theta [kg/m**3] (Calculated according to UNESC…)	kg m^−3^	27
Dissolved oxygen	O2 [µmol/l] (Dissolved Oxygen Sensor, Sea-…)	μmol L^−1^	27
Dissolved oxygen saturation	O2 sat [%] (Calculated according to UNESC…)	%	27
Photosynthetically active radiation	PAR [µE/m**2/s] (PAR sensor, Biospherical, LI-…)	Photons m^−2^ s^−1^	19
Turbidity	beta700 [m/sr] (Scattering meter, WET Labs, E…)	m sr^−1^	20
Attenuation	Attenuation [1/m] (Transmissometer, WET Labs, C-…)	m^−1^	20
Nitrate	[NO3]- [µmol/l] (Colorimetric analysis)	μmol L^−1^	15
Phosphate	[PO4]3- [µmol/l] (Colorimetric analysis)	μmol L^−1^	15
Silicate	Si(OH)4 [µmol/l] (Colorimetric analysis)	μmol L^−1^	15
Particulate organic carbon	POC [µmol/l] (Organic Elemental Analyzer, T…)	mg C m^−3^	13
Dissipation 1	Diss rate [W/kg] (shear 150–300 m and strain 20…)	W kg^−1^	14
Diffusivity 1	K rho [m**2/s] (shear 150–300 m and strain 20…)	m^−2^ s^−1^	14
Dissipation 2	Diss rate [W/kg] (shear 70–200 m and strain 30-…)	W kg^−1^	14
Diffusivity 2	K rho [m**2/s] (shear 70–200 m and strain 30-…)	m^−2^ s^−1^	14
Chlorophyll-a	Chl a [mg/m**3] (Fluorometer, Chelsea Instrume…)	mg m^−3^	27
Net primary productivity	NPP C [mmol/m**3/day] (Liquid scintillation counter,…)	mmol C m^−3^ d^−1^	8
Dissolved organic carbon	DOC [µmol/l] (High Temperature Catalytic Ox…)	μmol L^−1^	1
DOC flux	DOC flux [mg/m**2/day] (Calculated)	mg C m^−2^ d^−1^	1
Ambient leucine assimilation	Leu upt rate [pmol/l/h] (Radioassays, liquid scintilla…)	pmol L^−1^ h^−1^	6
Bacterial cell count	Bact [#/ml] (Flow cytometer, Becton Dickin…)	cells mL^−1^	6

**Table 2:** Parameters included in ship-based meteorlogical data file.

Parameter	Column header	Units
DateTime	Date/Time	YYYY-MM-DD HH:mm:ss
Latitude	Latitude	degrees North
Longitude	Longitude	degrees East
Altitude	Altitude [m]	metres
Air pressure	PPPP [hPa] (Barometer, Vaisala, PTB 210)	mBar
Air temperature	TTT [°C] (Temperature and humidity sens…)	degrees celsius
Air humidity	RH [%] (Temperature and humidity sens…)	%
Port solar radiation	PISR [W/m**2] (port, Pyranometer, Kipp & Zon…)	W m^−2^
Starboard solar radiation	PISR [W/m**2] (starboard, Pyranometer, Kipp…)	W m^−2^
Port surface photosynthetically active radiation	PAR [W/m**2] (port, PAR sensor, Two Skye In…)	W m^−2^
Starboard surface photosynthetically active radiation	PAR [W/m**2] (starboard, PAR sensor, Two Sk…)	W m^−2^

**Table 3:** Parameters included in Discrete POC concentration and flux data file.

Parameter	Column header	Unit
Event	Event	NA
DateTime	Date/Time	YYYY-MM-DD HH:mm:ss
Latitude	Latitude	degrees North
Longitude	Longitude	degrees East
Site	Site	P3A, P3B, P3C
Depth	Depth water [m]	metres
Particulate organic carbon concentration	POC [mg/m**3]	mg C m^−3^
Fast MSC particulate organic carbon flux	POC flux [mg/m**2/day] (fast, Marine snow catcher)	mg C m^−2^ d^−1^
Slow MSC particulate organic carbon flux	POC flux [mg/m**2/day] (slow, Marine snow catcher)	mg C m^−2^ d^−1^
Total MSC particulate organic carbon flux	POC flux [mg/m**2/day] (total, Marine snow catcher)	mg C m^−2^ d^−1^

**Fig. 2:**
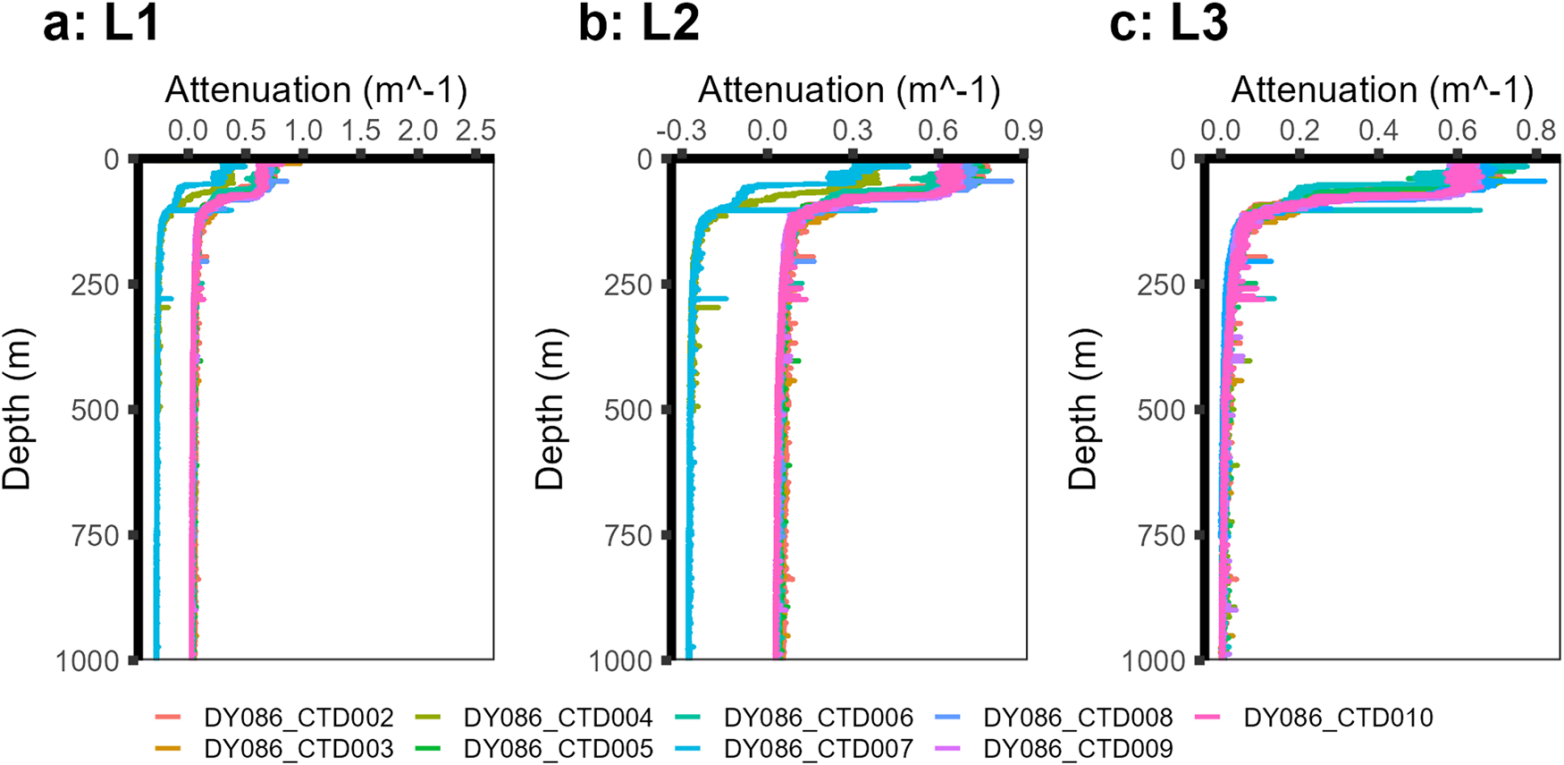
Levels of processing and data cleaning in attenuation profiles from P3A displaying **(a)** raw data, **(b)** the removal of the CTD ‘dip’ (b) which clearly demonstrates the difference in data between the titanium (CTD004 and CTD007) and stainless steel (all others) rosettes, and **(c)** all profiles with deepest values subtracted to normalise the data between rosettes.

This work includes newly processed biogeochemical parameters PAR, turbidity and attenuation. PAR is presented as the mean of one-metre bins of raw downcast data (night-time profiles are included). Beam attenuation was calculated from factory-calibrated transmittance. Raw turbidity and attenuation data underwent the following: upcast removal and removal of CTD ‘dip’ data so that profiles begin at 5 metres on the downcast. Further, attenuation data showed a consistent divergence in signal between the two rosettes (Fig. 2a,b). To correct for this, a ‘deep blank’ was calculated for each profile and subtracted (Fig. 2c). The deep blank was set to a minimum value between the deepest 50 metres of a profile. However, profiles where the maximum depth was less than 600 metres were removed as the signal had not yet stabilised; data points below 1000 metres were removed as the focus of this dataset is the biological carbon pump through the mesopelagic region. Data were then binned onto 1-metre intervals.

DateTime, Latitude and Longitude columns were added to files ‘*Ship-based meteorological data’*, ‘*Discrete POC concentration and flux data*’, ‘*Net-derived biomass data*’ (Table 4) and ‘*Acoustic backscatter data*’ (Table 5). *Discrete POC concentration and flux data* contains discrete ship-based measurements of POC and POC flux; these data were used to calibrate the simultaneous glider backscatter data collected as part of the GOCART project^11,15,16^. Versions of *Discrete POC concentration and flux data* were previously available on request from the author^11^ but have now been made instantly accessible, and event numbers were included. *Net-derived biomass data* constitutes a temporal average but first and last Event numbers from the ship’s Event Log were included for each data value. Data included in *Net-derived biomass data* were provided by Dr Kathryn Cook. *Net-derived biomass data* constitutes a summary of active flux values relevant for BCP investigation that are plotted in Figs. 2,4,5 (*pages 7, 8 and 9, respectively*)^17^. For *Acoustic backscatter data*, raw acoustic data were provided by Dr Sophie Fielding. The depth-zonal means of these data are described in the literature and plotted in Figures S1, S2 on Pages 7-8 of their Supplementary Data^17^, but the data remained unpublished prior to this work. Code containing the required analysis to produce their Supplementary Figures S1, S2 could not be made available. As such, any mean Sv values less than -100 decibels were removed before separating into day and night values. Then, using smooth.spline from R’s ‘stats’ package (version 3.6.2) with 10 degrees of freedom to recreate data in the plots, a new column was created on 10-metre depth bins for each frequency.

**Table 4:** Parameters included in net-derived biomass data file.

Parameter	Column header	Unit
Event	Event	NA
Latitude	Latitude	degrees North
Longitude	Longitude	degrees East
DateTime	Date/Time	YYYY-MM-DD HH:mm:ss
Site	Site	P3A, P3B, P3C
First event number	Run [#] (initial)	NA
Last event number	Run [#] (final)	NA
Event count	Runs [#]	NA
Day/night	Time of day	d/n
Depth mean	Depth water [m] (Mean values)	metres
Depth upper	Depth water top [m]	/
Depth lower	Depth water bot [m]	metres
Zooplankton biomass	Zoopl micronekton C [mmol/m**3] (Calculated)	mmol C m^−3^
Zooplankton respiration	Zoopl micronekton resp C [mmol/m**3/day] (Calculated)	mmol C m^−3^ d^−1^
Zooplankton ingestion	Zoopl micronekton IR C [mmol/m**3/day] (Calculated)	mmol C m^−3^ d^−1^

**Table 5:** Parameters included in acoustic backscatter data file.

Parameter	Column header	Unit
Event	Event	NA
DateTimeStart	Date/Time	YYYY-MM-DD HH:mm:ss
DateTimeEnd	Date/Time 2	YYYY-MM-DD HH:mm:ss
Latitude	Latitude	degrees North
Longitude	Longitude	degrees East
Site	Site	N/A
Frequency	Frequency [kHz]	kHz
Day/night	Time of day	‘d’ or ‘n’
Depth	Depth water [m]	metres
Backscatter	Backsc [dB]	decibels

## Data Records

The dataset is available at PANGAEA^18–22^. PANGAEA follows FAIR data principles; in particular, data is more findable than comparable repositories. The fields for each data file are included below. A citation is included for data that have been described previously.

### Ship-based CTD profile data (Major-etal_2023_CTD)

Data collected via sensors attached to the CTD rosette and subsequent bottle data analysis^18^. Data were averaged into 1-metre depth bins.

### Ship-based meteorological data (Major-etal_2023_meteorology)

Meteorological data collected by ship-fitted systems; a reading was provided for every minute at each site^19^.

### Discrete POC concentration and flux data (Major-etal_2023_POC_disc)

Discrete instrument data used to determine POC concentrations and calculate POC fluxes including MSCs, SAPS and PELAGRA Traps^20^.

### Net-derived biomass data (Major-etal_2023_biomass)

Discrete net-derived data containing biomass, respiration and ingestion calculations^17,21^.

### Acoustic backscatter data (Major-etal_2023_Sv)

Acoustic backscatter data in five frequencies (18, 38, 70, 120 and 200 kHz) separated into day and night profiles^22^. Backscatter profiles were averaged across each site visit^17^. First and last event numbers and event count (total deployments) for each data point are included.

## Technical Validation

**Fig. 3:**
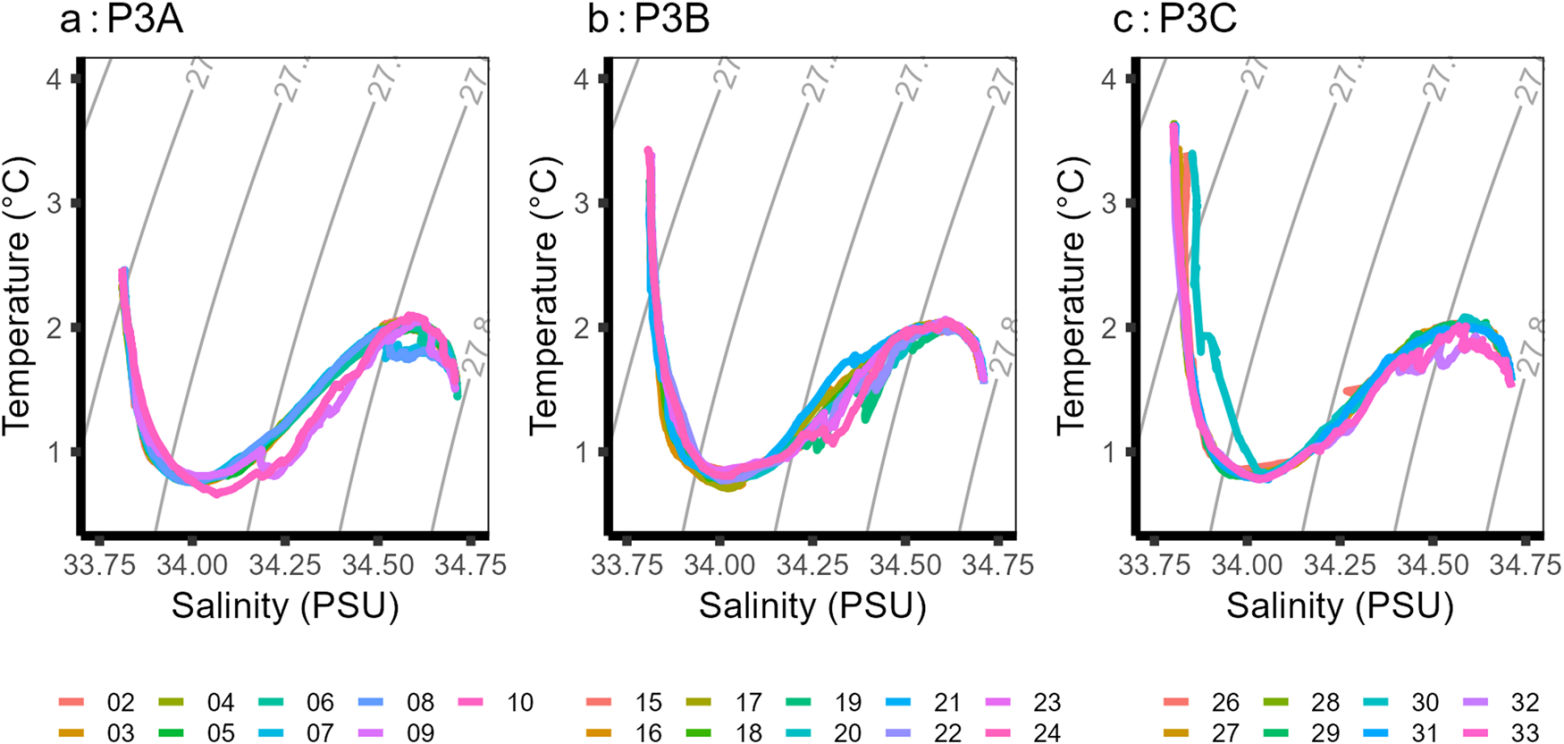
Temperature-salinity plots across the three P3 site visits for individual CTD profiles. Grey lines denote Sigma-Theta density intervals. CTD profiles 1, 11, 12, 13, 14 and were taken at non-P3 sites and are thus not included.

Data presented here achieve technical validation because all sensors were calibrated within the timescale recommended by manufacturers prior to deployment (see file ‘parameters_instruments_methods.csv‘ for calibration dates), expert knowledge went into data collection, and data have been plotted and visually checked for consistency (e.g. Figure 3). On top of this, much of the data has been described elsewhere and has successfully undergone the scientific review process. Water samples collected from the sample bottles were taken using standard best practices and methods are outlined in the cruise report^23^ and in the aforementioned literature. Further, methods for newly presented data PAR, turbidity and attenuation have been outlined and exemplified in Fig. 2.

**Fig. 4:**
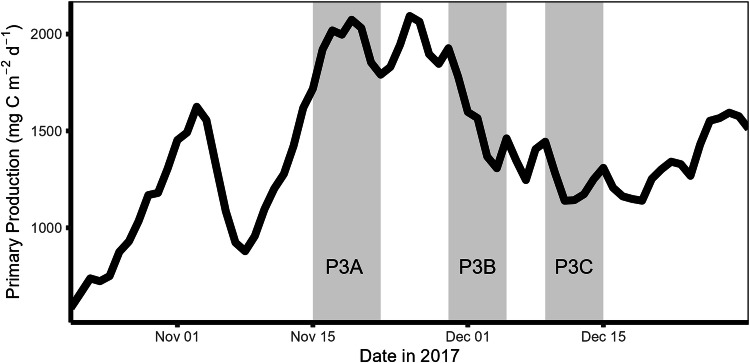
Satellite primary productivity (mg C m^−2^ d^−1^) at site P3 across November and December, 2017, with the three visits to the site made by RRS Discovery highlighted in grey.

**Fig. 5:**
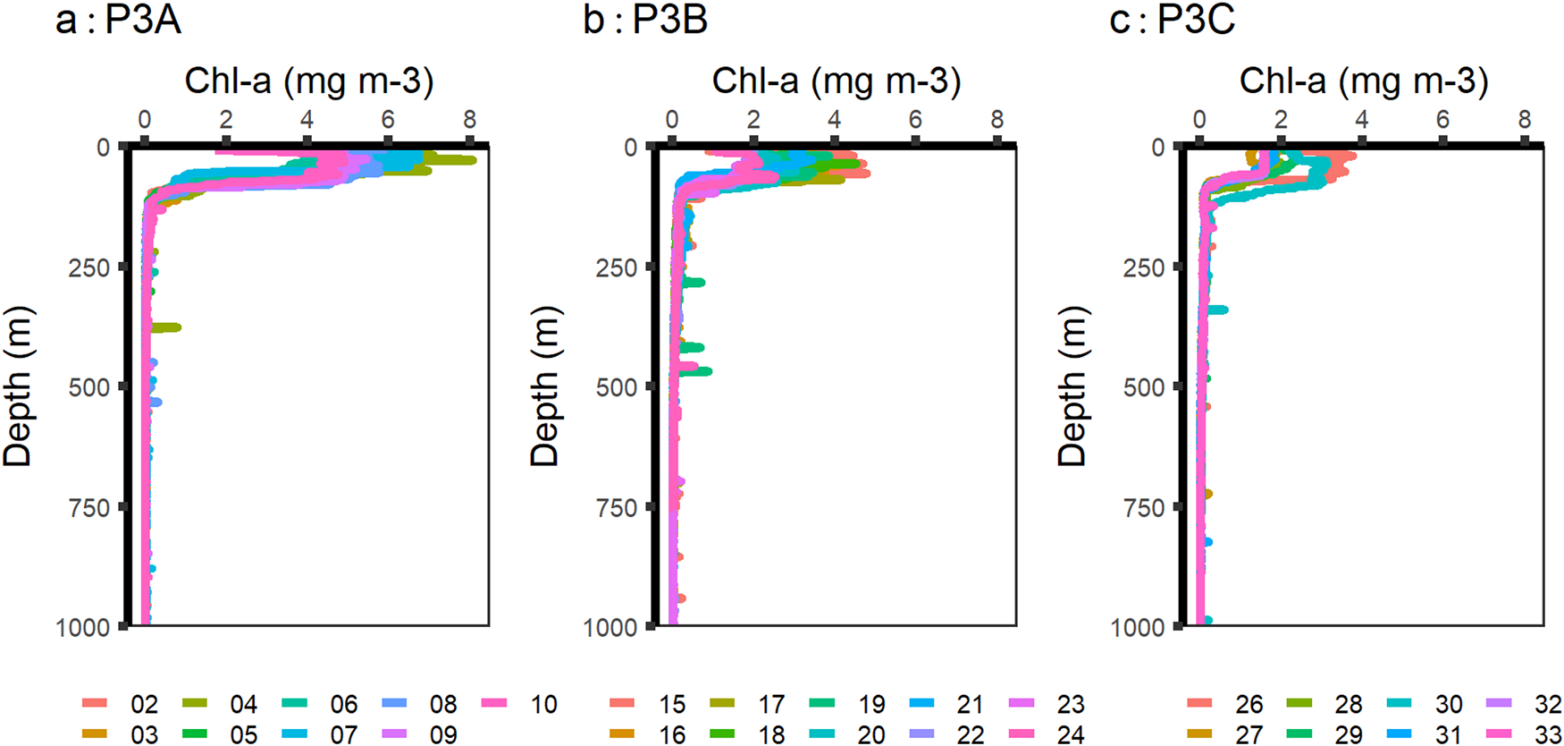
Chlorophyll-a plots across the three P3 site visits for individual CTD profiles. There is a decreasing trend in surface-level chlorophyll-a from P3A to P3C as the austral spring bloom subsides.

Available ship-based measurements are consistent with satellite data. Satellite data suggests the peak austral spring bloom (2093.1 mg C m^−2^ d^−1^) occurred over the course of the cruise (Fig. 4). Chlorophyll-a measurements presented here correlate with satellite measurements throughout the field campaign and demonstrate the decline in the bloom (Fig. 5). However, there are limitations of the POC concentration and flux data: PELAGRA traps may under sample small particles due to their conical shape^24^; MSCs may not represent the study site as a whole as they are instantaneous snapshots^15^; POC bottle and in situ pump data also come with accuracy complications^25^. Glider-derived backscatter generally represented the spread of POC concentration and flux data^11^ and was used to generate high-resolution POC data that have also been made available with PANGAEA^16^. For DOC data, the single profile did not permit statistical analysis of concentration and flux but these were consistent with other data collected in the region^12^. Moreover, uncertainties in DOC flux estimates are unlikely to impact the overall interpretation of the study site as diapycnal DOC flux contributed <0.1% to overall carbon flux during data collection, which was dominated by gravitational flux^13^.

**Fig. 6:**
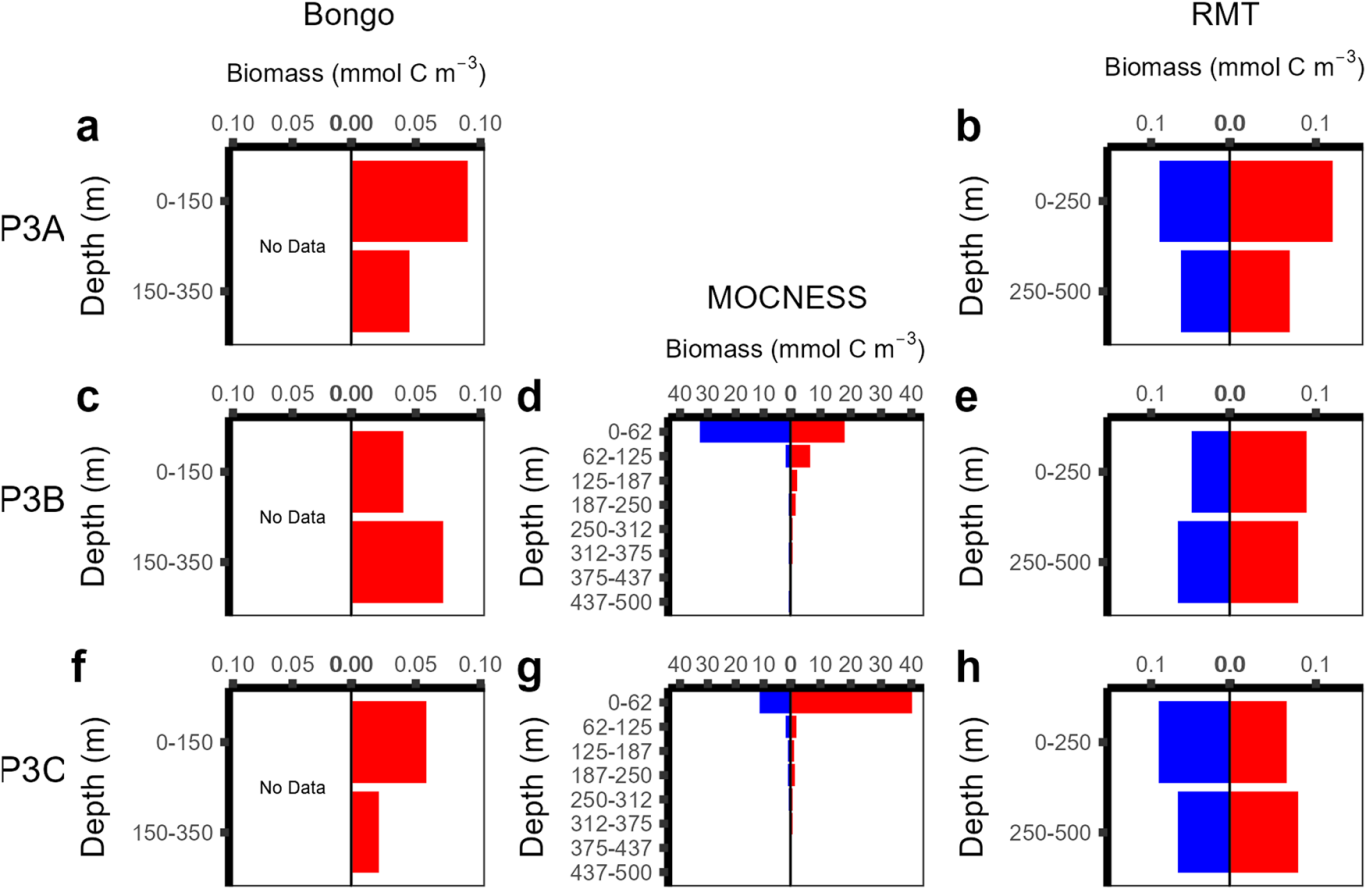
Profiles of zooplankton biomass. Zooplankton biomass was collected with Bongo (**a,c,f**), MOCNESS (**d,g**) and RMT25 (**b,e,h**) across the three P3 site visits. MOCNESS and RMT25 nets were deployed at night (blue) and day (red), but Bongo was deployed during daytime only. MOCNESS was not deployed at P3A. Reproduced after^17^.

**Fig. 7:**
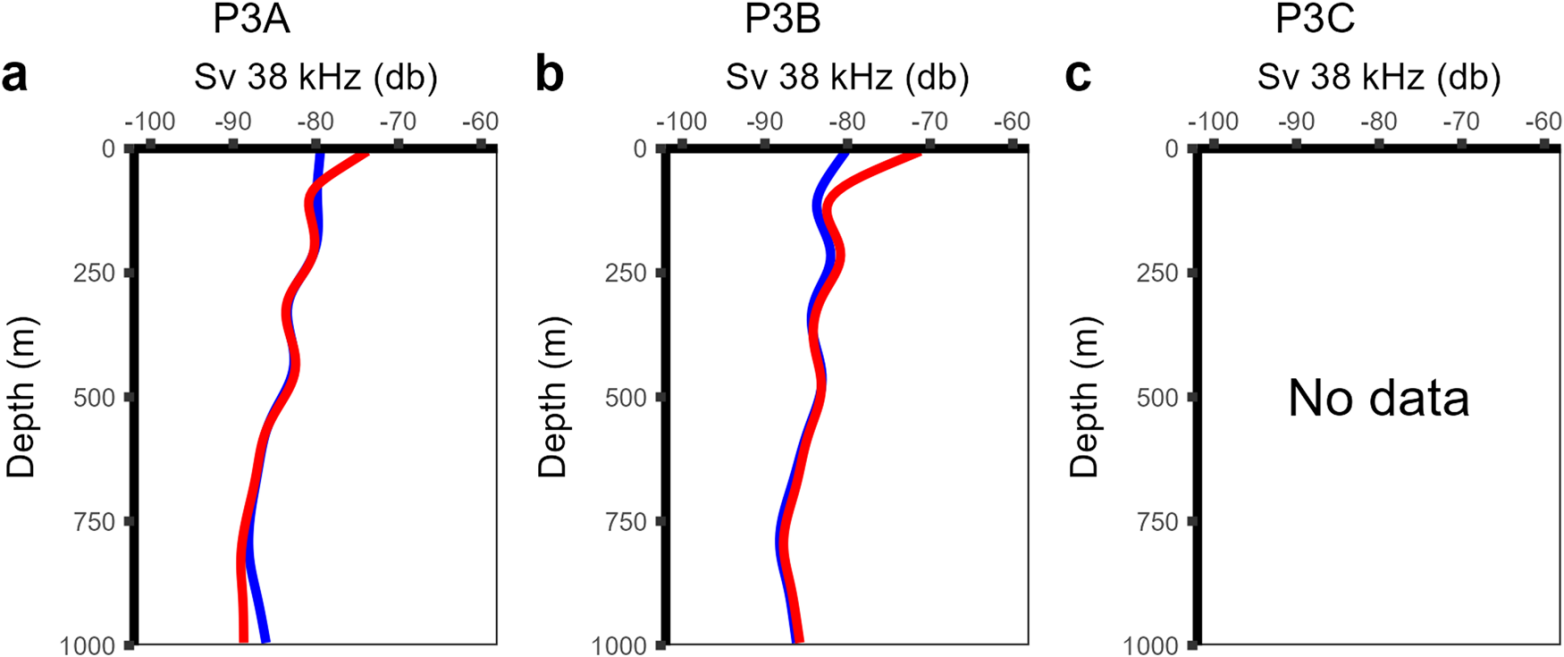
Acoustic backscatter profiles at 38 kHz across two P3 site visits. Data were separated into night (blue) and day (red). Acoustic backscatter consistently shows little to no evidence of diel vertical migration. No acoustic data were available for site visit P3C.

Data on active flux had several limitations: time limits meant that Bongo nets were not deployed during night-time hours and MOCNESS was not deployed at P3A^17^. Hence, diel vertically migrating copepods may not have been captured due to the lack of night-time Bongo net deployments. Furthermore, it is possible that organisms vertically migrated to depths greater than 500 metres; nets were deployed to a maximum of 500 metres depth. While diel vertical migration was observed in some species^17^, there was no consistent evidence of synchronised diel vertical migration in net-collected biomass data (Fig. 6). In line with observations of no diel vertical migration, day and night acoustic backscatter data (Fig. 7) supported the lack of evidence of synchronous migration between the surface and 1000 metres depth^17^. However, any active flux generated through asynchronous vertical migration is not detectable by standard acoustics and net sampling. The use of a bi-directional net from a nearby study site that elucidates asynchronous migration suggests active flux may be underestimated in this dataset^26^.

Dissolved oxygen saturation data appear to be elevated nearer the surface relative to other data sources (e.g. ~110% in this study compared with ~100% from GLODAP data^27^). Bottle oxygen data from this study show reasonable agreement with GLODAP data. However, calibrated dissolved oxygen sensor data presented here show greater variation nearer the surface when compared with bottle measurements. Therefore, we recommend that caution is applied to findings that make use of dissolved oxygen saturation data from near the surface.

## Usage Notes

These data can be used by observational scientists and modellers to investigate the processes contributing to organic carbon and related ecosystem interactions. The data can be used to further elucidate the effect of a phytoplankton bloom on the efficiency of the BCP. The study site P3 is characterised by elevated iron concentrations and low current speeds; hence, caution must be taken when applying findings derived from these data to different regions of the ocean.

We highly recommend making use of the following: high-resolution glider-derived backscatter POC concentration and flux data from the GOCART project that has been calibrated using ship-based measurements made during this cruise^16^; the BODC repository for physical, biogeochemical, meteorological parameters along with the cruise report (https://www.bodc.ac.uk/resources/inventories/cruise_inventory/report/16383/); ETS-derived respiration rates for micronekton and zooplankton from BODC (10.5285/b9f5c5ec-100a-7ff0-e053-6c86abc0f494)^17^; Rectangular Midwater Trawl net catch data from British Antarctic Survey (https://data.bas.ac.uk/full-record.php?id=GB/NERC/BAS/PDC/01337)^17^.

## Data Availability

No custom code was used to produce data. All code used to synthesise and analyse data is available on GitHub: https://github.com/obg-wrm/COMICS_data.
